# Advantages of gadolinium based ultrasmall nanoparticles vs molecular gadolinium chelates for radiotherapy guided by MRI for glioma treatment

**DOI:** 10.1186/s12645-014-0004-8

**Published:** 2014-07-01

**Authors:** Géraldine Le Duc, Stéphane Roux, Amandine Paruta-Tuarez, Sandrine Dufort, Elke Brauer, Arthur Marais, Charles Truillet, Lucie Sancey, Pascal Perriat, François Lux, Olivier Tillement

**Affiliations:** ID17 Biomedical Beamline, European Synchrotron Radiation Facility, 6 rue Jules Horowitz, 38000 Grenoble, France; Institut UTINAM, UMR 6213 UFC-CNRS, Université de Franche-Comté, 16 route de Gray, 25030 Besançon, Cedex France; Institut Lumière Matière, UMR 5306 Université Lyon 1 – CNRS, Team FENNEC, Université de Lyon, 69622 Villeurbanne, Cedex France; Nano-H S.A.S, 2 Place de l’Europe, 38070 Saint Quentin-Fallavier, France; MATEIS, UMR 5510 INSA Lyon – CNRS, INSA Lyon, 69621 Villeurbanne, France

**Keywords:** 3–10 : Gadolinium, MRI, DOTA, Radiosensitization, Nanoparticles

## Abstract

AGuIX nanoparticles are formed of a polysiloxane network surrounded by gadolinium chelates. They present several characteristics. They are easy to produce, they present very small hydrodynamic diameters (<5 nm) and they are biodegradable through hydrolysis of siloxane bonds. Such degradation was evaluated in diluted conditions at physiological pH by dynamic light scattering and relaxometry. AGuIX nanoparticles are also known as positive contrast agents and efficient radiosensitizers. The aim of this paper is to compare their efficiency for magnetic resonance imaging and radiosensitization to those of the commercial gadolinium based molecular agent: DOTAREM®. An experiment with healthy animals was conducted and the MRI pictures we obtained show a better contrast with the AguIX compared to the DOTAREM® for the same amount of injected gadolinium in the animal. The better contrast obtained after injection of Aguix than DOTAREM® is due to a higher longitudinal relaxivity and a residential time in the blood circulation that is two times higher. A fast and large increase in the contrast is also observed by MRI after an intravenous injection of the AGuIX in 9 L gliosarcoma bearing rats, and a plateau is reached seven minutes after the injection.

We established a radiotherapy protocol consisting of an irradiation by microbeam radiation therapy 20 minutes after the injection of a specific quantity of gadolinium. After microbeam radiation therapy, no notable difference in median survival time was observed in the presence or absence of gadolinium chelates (38 and 44 days respectively). In comparison, the median survival time is increased to 102.5 days with AGuIX particles showing their interest in this nanomedicine protocol. This remarkable radiosensitizing effect could be explained by the persistent tumor uptake of the particles, inducing a significant nanoscale dose deposition under irradiation.

## Background

As emphasized by J. N. Long *et al.* in a recent review [1], molecular gadolinium chelates and in particular those involving 1,4,7,10-tetraazacyclododecane-1,4,7,10-tetraaccetic acid (DOTA) have been extensively developed and used in biomedical imaging for the last 25 years. First used for magnetic resonance imaging (MRI), DOTA(Gd^3+^) (commercially known as DOTAREM®) offers the advantage of a better thermodynamic association constant as well as lower dissociation kinetic rates in comparison to acyclic ligands [2]. These characteristics are essential to safely use a gadolinium-based contrast agent and avoid nephrogenic systemic fibrosis (NFS) [3] that might be induced by the *in vivo* demetallation of gadolinium*.* DOTA and DOTA derivatives can also be used for the chelation of radioactive isotopes (*e.g.*^111^In or ^64^Cu) to perform single photon emission computed tomography (SPECT) [4] or positron emission tomography (PET) [5]. They can be coupled to various types of targeting ligands (*e.g.* antibodies [6], peptides [7], aptamers [8]) to render the imaging more sensitive and efficient. In line with the recent development of fused instruments to perform multimodal imaging (PET/MRI (Magnetic Resonance Imaging), SPECT/CT or PET/CT (computed tomography), Fluorescence/MRI) [9], DOTA based Gd^3+^ complexes can be associated with other imaging agents, such as radionuclide chelators or organic luminescent probes [10–12].

Another strategy to enhance the imaging contrast and to permit multimodal imaging as well as theranostic applications has been proposed recently. It consists in coupling the chelating species to the surface of nanoparticles [13,14], or to incorporate them into nanostructures [15]. As MRI suffers from low sensitivity [16], coupling gadolinium chelates to nanostructures is an advantage for MRI because it enhances the longitudinal relaxivity both per object (by grafting multiple chelates on one object) and per gadolinium (by increasing the rotational correlation time) [17]. Another advantage of using nanoparticles is that they do not present any extravasation from normal vessels and are accumulated passively in tumors *via* the enhanced permeability and retention (EPR) effect [18]. Finally, the use of efficient MRI contrast agents provides a better delineation of the tumor and consequently a better image guidance for further radiotherapy protocol. The clinical interest for image guided radiation therapy (IGRT) is for example emphasized by the partnership between Elekta (radiotherapy systems manufacturers) and Philips (medical imaging specialists) [19]. In this context, gadolinium is playing an important role because of its intrinsically properties for MRI and because of its relatively high atomic number value (Z = 64). This is important since many molecules [20] containing bromine (Z = 35), iodide (Z = 53), gadolinium or platinum (Z = 78) or nanoparticles [21] made with gold (Z = 79) have shown great potential in radiosensitization. Since the pioneering work of Hainfeld *et al.* [21] a large number of experimental reports using particles with different photon source energies as well as different cell lines confirms the interest in nanoparticles to enhance the clinical irradiation efficiency [22,23]. For the moment, the most studied gadolinium-based radiosensitizer is Motexafin Gadolinium (MGd). It contains a porphyrin like macrocycle that chelates gadolinium. MGd can be detected by MRI and is currently in the phase III of clinical development as a radiosensitizer for irradiation of brain tumors [24–26]. As theoretical work and experiments suggest that heavy elements in nanoparticles improve radiosensitization [22], our team has developed a family of ultrasmall gadolinium based nanoparticle for MRI and radiosensitization called AGuIX (Activation Guided by Irradiation by X-rays). Two main types of nanoparticles were proposed that present very similar *in vivo* behaviors. The first nanoparticles consist of a polysiloxane core coated with DTPA (Diethylene Triamine Pentaacetic Acid) [27] and have demonstrated their potential for IGRT. To completely prevent any release of gadolinium, a new generation of nanoparticles incorporating DOTA was proposed [28,29]. For their synthesis, a DOTA derivative: DOTAGA [30,31] (GA = Glutaric Acid) was used for the grafting on the nanoparticle. The additional arm of this macrocycle is used to form an amide bond leaves eight coordination atoms for the chelation of gadolinium as in molecular DOTA. This coordination mode leads to a similar association constant towards gadolinium for the DOTAGA grafted on the nanoparticle compared to molecular DOTA towards gadolinium (log β = 24.78 and 25.58 respectively) [29]. Interestingly, the two types of nanoparticles have very similar physico-chemical properties, radiosensitizing properties and *in vivo* biodistribution profiles. The aim of this paper is to demonstrate the interest of these nanoparticles supporting gadolinium chelates for the treatment of glioma by IGRT compared to using small gadolinium chelates.

## Methods

### Chemicals

Gadolinium chloride hexahydrate ([GdCl_3_, 6H_2_O], 99.999%), sodium hydroxide (NaOH, 99.99%), tetraethyl orthosilicate (Si(OC_2_H_5_)_4_, TEOS, 98%), aminopropyl-triethoxysilane (H_2_N(CH_2_)_3_-Si(OC_2_H_5_)_3_, APTES, 99%), triethylamine (TEA, 99.5%), 4-(2-hydroxyethyl)-1-piperazineethanesulfonic acid (HEPES, 99.5%), sodium hydroxide (NaOH, 99.99%), sodium chloride (NaCl, 99.8%), calcium chloride (CaCl_2_, 99%), the bovine serum albumin and dimethyl sulfoxide (DMSO, 99.5%) were purchased from Aldrich Chemicals (France). Diethylene glycol (DEG, 99%) was purchased from SDS Carlo Erba (France). Acetone (reagent grade) was purchased from Sodipro (France) and was used in the same conditions as received. 1,4,7,10-tetraazacyclododecane-1-glutaric anhydride-4,7,10-triacetic acid (DOTAGA) was furnished by CheMatech (Dijon, France). Gadolinium oxide cores were furnished by Nano-H S.A.S (Saint-Quentin Fallavier, France). Only milli-Q water was used for the preparation of aqueous solutions of nanoparticles.

### Characterization

#### DLS (Dynamic Light Scattering) size measurement

Direct measurements of the size distribution (in DEG) or after dilution (at 10 mM in [Gd^3+^] for water) of the nanoparticles were performed *via* Zetasizer NanoS DLS (laser He-Ne 633 nm) from Malvern Instrument.

#### ζ-potential measurements

Direct determination of the *ζ*-potential of the hybrid nanoparticles were performed *via* a Zetasizer NanoS from Malvern Instruments. Prior to the experiment, the nanoparticles were diluted in an aqueous solution containing 0.005 M in NaCl and adjusted to the desired pH.

#### Inductively coupled plasma-atomic emission spectrometry (ICP-AES) analysis

Determination of the gadolinium content in a sample was performed by ICP-AES analysis (with a Varian 710-ES spectrometer). Before measuring gadolinium concentration, samples of colloidal solution were dissolved in concentrated nitric acid for 24 hours. The samples were then diluted with water, until the nitric acid concentration in water reached 5%. Chemical analyses were also performed on the as-prepared samples at the Service Central d’Analyses du CNRS (Solaize, France) by ICP-AES, and enabled determining the C, N, Si contents with a precision of 0.5%.

#### Relaxometry

Relaxation time measurements were performed using a Bruker Minispec MQ60 NMR analyser, operating at 1.4 T magnetic field.

#### High performance liquid chromatography (HPLC)

Gradient HPLC analysis was done by using Shimadzu Prominence series UFLC system with a CBM-20A controller bus module, a LC-20 AD liquid chromatograph, a CTO-20A column oven and a SPD-20A UV-visible detector. UV-visible absorption was measured at 295 nm. 20 μL of sample were loaded in the solvent injection ratio: 95% solvent A – 5% solvent B (A = Milli-Q water/TFA 99.9:0.1 v/v; B = CH_3_CN/Milli Q water/TFA 90:9.9:0.1 v/v/v) onto a Jupiter C4 column (150 × 4.60 mm, 5 μm, 300 Å, Phenomenex) at a flow rate of 1 mL/min over 5 min. In a second step, samples were eluted by a gradient developed from 5 to 90% of solvent B in solvent A over 15 min. The concentration of solvent B was maintained over 5 min. Then, the concentration of solvent B was decreased to 5% over a period of 5 min to re-equilibrate the system, followed by additional 5 min at this final concentration. Before each sample measurement, a baseline was performed following the same conditions by loading Milli-Q water into the injection loop.

### Particles synthesis

#### Preparation of gadolinium oxide cores [32]

A solution was prepared by dissolving 167.3 g of [GdCl_3_, 6 H_2_O] in 3 L of DEG at room temperature. The solution is then stirred for 3 hours at 140°C. Afterwards, 44.5 mL of sodium hydroxide solution (10 M) is added to the solution. This final solution is stirred for 5 hours at 180°C before cooling and stirring for 12 hours at ambient temperature. The gadolinium oxide cores displayed hydrodynamic diameters of 1.7 ± 0.5 nm.

#### Encapsulation of gadolinium oxide cores by polysiloxane

The polysiloxane shell is ensured by sol–gel process by addition of silane precursors. First, a solution containing 1.6 L of DEG, 51.42 mL of TEOS and 80.61 mL of APTES is slowly added (during 96 hours) to the precedent solution under stirring at 40°C. One hour after the end of the addition of the silane precursors, a second solution of 190 mL of DEG, 43.1 mL of water and 6.94 mL of TEA is added under stirring at 40°C during 96 hours. At the end of the second addition, the solution is stirred for 72 hours at 40°C and finally for 12 hours at ambient temperature. The gadolinium oxide cores coated by the polysiloxane displayed hydrodynamic diameter of 2.6 ± 1.0 nm.

#### Covalent grafting of DOTAGA on the nanoparticles

163.7 g of DOTAGA anhydride are then added to the core-shell nanoparticles in DEG. The resulting solution is stirred for 72 hours at room temperature.

#### Purification

17.5 L of acetone are added to the solution to precipitate the nanoparticles that are filtered under vacuum before dispersion in water. The solution is stirred for one hour at pH 2 before remaining acetone is removed by evaporation. The nanoparticles are then purified by tangential filtration through 5 kDa membrane. The solution is stirred at pH 5 for 12 hours before another tangential filtration through 5 kDa membrane after addition of sodium hydroxide solution to reach pH 7.4. Afterward, the solution is filtered twice through a 1.2 μm and then 0.2 μm syringe filters to remove the largest impurities. Finally, the nanoparticles are freeze-dried and can be stored for months without alterations. After dispersion in water, the AGuIX nanoparticles display hydrodynamic diameter of 2.2 ± 1 nm. Elementary analysis found (weight percent) C, 26.84; N, 7.79; Gd, 13.83; Si, 10.79. An average mass of 8.7 ± 1 kDa is obtained by mass spectrometry. The longitudinal relaxivity (r_1_) is 11.5 mM^−1^.s^−1^ at 1.4 T. The nanoparticles display an isoelectric point at pH 7.5 ± 0.5. About 50 g of nanoparticles are obtained at the end of the synthesis. HPLC chromatogram, with UV absorption detection at 295 nm, shows a retention time of 10 min for the nanoparticles.

### Imaging and MRT procedure

All operative procedures related to experiments on glioma bearing rats strictly conformed to the French government regulations with licenses 380825 and B3818510002 and were approved by the internal evaluation committee for animal welfare and rights of the ESRF. All operative procedures related to MR on healthy mice strictly conformed to the Guidelines of the French Government with licenses 380324 and A3818510002 for MRI on healthy mice. All experiments were performed under anesthesia with the following parameters: 5% isoflurane for induction and intraperitoneal injection of xylazine/ketamine (64.5/5.4 mg.kg^−1^) for maintenance.

#### Brain tumor inoculation

The 9 L gliosarcoma (9LGS) cells were implanted in the brain of male fisher F344 rats (Charles River, France) [33]. Anesthetized animals were placed on stereotactic frame, and 10^4^ 9LGS cells were suspended in 1 μL culture medium with antibiotics before to be injected through a burr hole in the right caudate nucleus (3.5 mm lateral to the bregma, 6 mm below the skull surface).

#### Preparation of injectable solution

After tangential filtration, a concentrated colloid (AGuIX in water, [Gd^3+^] = 100 mM) was diluted by aqueous solution containing NaCl and hepes in order to obtain an intravenous use solution ([Gd^3+^] = 40 mM, [NaCl] = 145 mM, [hepes] = 10 mM). The pH was adjusted to 7.4. Before use, this solution was filtered onto syringe filter with nylon membrane (pore diameter 0.22 μm). The chelate used was DOTAREM® (laboratories Guerbet, Aulnay sous Bois France, 0,5 mM/mL) as available in MRI units.

#### Drug injection

The aqueous AGuIX ([Gd^3+^] = 40 mM, [NaCl] = 145 mM, [hepes] = 10 mM) colloid was manually injected in the saphena vein at 1.4 mL volume using a 2 mL syringe and a 26 G needle. The gadolinium chelates were injected via the saphena vein according 2 protocols. First, a series of rats received an injection of a colloidal solution at the concentration of 1 M in Gd , i.e. twice the concentration routinely used in clinical conditions (0.5 mM). The volume injected was of 56 μL which corresponds to a quantity of Gd (56 μmol) similar to those currently injected. Second, another series of rats received a 1.4 mL DOTAREM® injection diluted in physiological serum until 40 mM (to establish a direct comparison with the nanoparticles, the quantity of Gd injected being the same than previously).

#### MR imaging

The MR imaging took place at Grenoble Institute of Neuroscience (GIN) using a 7 Tesla Imaging system (Biospec, Bruker, Erlangen Germany) equipped with a 400 mM/T gradient. The images were made 14 days after implantation, and the rats were injected in the saphena vein with 1.4 ml of a Gd based particles solution at 40 mM in gadolinium. Images were acquired at fixed times after injection using a T2 weighted Turbo RARE SE sequence (TR = 4,000 ms, TE = 33 ms, FOV = 3 cm, Resolution = 0,12 mm, ST = 1 mm) and a T1 weighted FLASH sequence (TR = 840 ms, TE = 10,804 ms, FOV = 3 cm, Resolution + 0,12 mm, ST = 1 mm).

#### Radiation source and microbeam radiation therapy (MRT) set-up

Irradiations were performed at the ID17 Biomedical Beamline of the European Synchrotron Radiation Facility (ESRF, France) using X-rays emitted tangentially from electron bunches circulating in a storage ring. The wiggler produces a wide spectrum of photons which extends, after filtration, from 50 over 350 keV (median energy: 90 keV). The mean dose rate was then 62 Gy.mA^−1^.s^−1^ allowing very fast irradiation. The quasilaminar beam was micro-fractionated into an array of 41 rectangular and quasi-parallel 50 microns width microbeams, separated by 200 microns centre to centre. The setup was performed by using the ESRF Multislit Collimator, positioned 33 m from the photon source, and 80 cm upstream from the rat holder. Ten days after tumor inoculation, the animals were positioned prone on a Kappa-type goniometer (Huber, Germany) in front of the X-rays source, on a homemade Plexiglas frame, and the alignment into the beam was performed using live cameras. The contention of the rats was performed by a teeth bar, while the animals were under anaesthesia. They were received a lateral irradiation, from their anatomical right to left side, followed by an antero-posterior irradiation (cross fired configuration). The beam was shaped into a field of irradiation of 10 mm horizontal, and the animals were scanned vertically over 10 mm through the beam after opening of the shutter. Although the total procedure lasted about 2 min, for each rat, the irradiation time is around 2 s. Animal immobility during exposure was checked on three control video screens located in the control hutch. The microbeam dose at the tumor (i.e. 7 mm of depth from lateral side) was 400Gy, the valley dose was 18.6 Gy as computed by Monte Carlo simulations. The spatial configuration of irradiation was checked by radiochromic films (Gafchromic, HD-810) exposed in front of rats.

#### Survival analysis

The survivals of animals were represented on Kaplan − Meier curves and compared using the log-rank test; the Median Survival time (MeST) postimplantation was calculated (Prism, GraphPad Software, San-Diego, USA).

## Results and discussion

### Synthesis

A protocol for the synthesis of AGuIX has been previously published [28,29]. A scale-up synthesis has been developed in order to produce larger quantities of nanoparticles for *in vivo* applications and further potential clinical applications. The first step is the formation of a gadolinium oxide core by a slow addition of NaOH solution on gadolinium trichloride previously dissolved in diethylene glycol at high temperature. The gadolinium oxide cores are then coated by a polysiloxane shell by addition of suitable silane precursors. DOTAGA anhydride is then added to the nanoparticles; the chelate is grafted by a peptide bond on the surface of the polysiloxane matrix. The dispersion of these nanoparticles in water leads to the dissolution of the gadolinium oxide core which is induced by the chelation of the gadolinium by the ligands. As a result, ultrasmall nanoparticles are obtained, displaying hydrodynamic diameter of 2.1 ± 1 nm and a mass of 8.7 ± 1 kDa [29]. They are composed of a polysiloxane core surrounded by about ten gadolinium chelates displaying hydrodynamic diameter of 2.1 ± 1 nm and a mass of 8.7 ± 1 kDa.

### Characterization of the biodegradation

**Figure 1:**
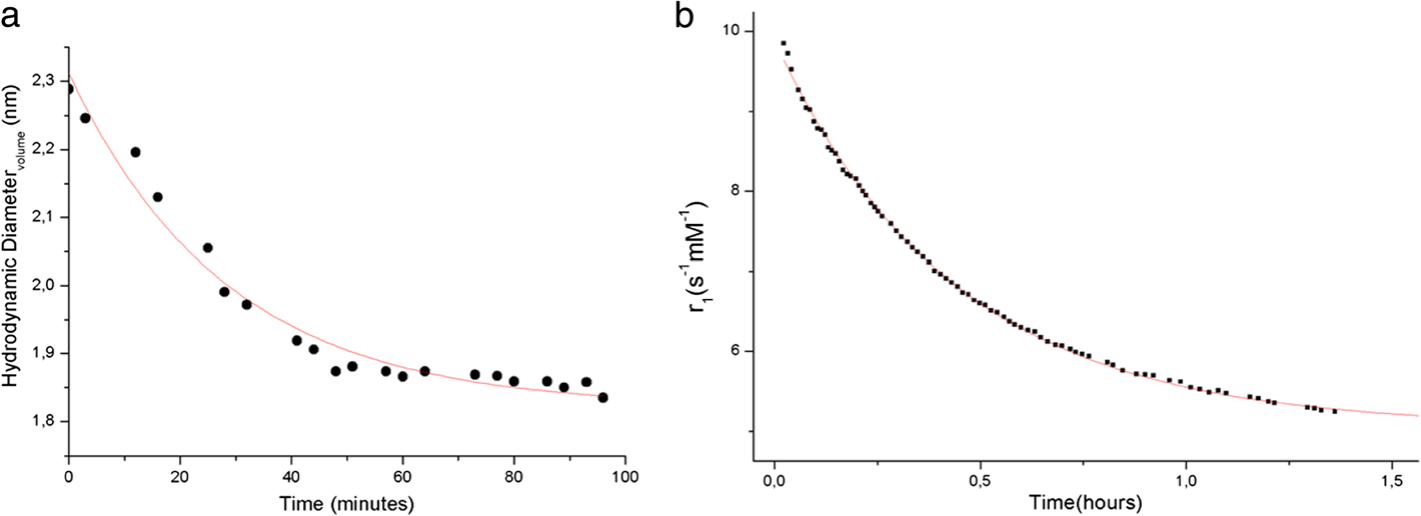
**Study of the degradation of the nanoparticle in diluted media after dilution at 2.5 mM. a)** Black circles correspond to temporal evolution of the hydrodynamic diameter. **b)** Temporal evolution of the longitudinal relaxivity. In the two cases, red lines correspond to monoexponential decay fit.

The nanoparticles present biodegradable properties in diluted or basic media. A cleavage of the oxygen-silicon bonds is observed in these conditions, leading to smaller nanoparticles that possess the same characteristics than the initial ones: they are also made of polysiloxane and surrounded by gadolinium chelates [34]. In order to characterize the dissolution process, the freeze-dried nanoparticles have been dispersed at 50 mM (in Gd^3+^) at physiological pH in a solution containing 0.1 M of sodium chloride and 1.5 mM of calcium chloride and stirred at this concentration for one hour to permit homogeneous dispersion of the nanoparticles. They are diluted ([Gd^3+^] = 2.5 mM) in a solution containing 0.1 M of sodium chloride and 1.5 mM of calcium chloride. The hydrodynamic diameter obtained by DLS has been plotted versus time for a temperature of 37°C (See Figure 1). The curve has been fitted by a mono-exponential decay curve and lead to a half-life of about 19.6 minutes. Moreover, the degradation can be followed by relaxometry thanks to the longitudinal relaxivity (r_1_), which is higher for greater nanoparticles mass due to an increase in the rotational correlation time [17]. Temporal evolution of r_1_ per gadolinium has been plotted after dilution at 2.5 mM in bovine serum albumin. This serum has the same main properties than human serum (See Figure 1). After fitting hydrodynamic diameter and relaxivity by a mono-exponential decay curve, a similar half-life of 17.9 minutes is observed, indicating a non-negligible degradation in diluted solutions close to *in vivo* conditions. The degradation life-time is close to the order of magnitude of the residence time, which may explain the observed efficiency of the renal elimination.

### MRI study

#### On healthy animals

The AGuIX nanoparticles have been intravenously injected (80 μL at 40 mM in Gd) on 8-weeks-old c57B1/6 J mice and have been followed by MRI at 7 T (r_1_ = 6 mmol^−1^.s^−1^) [28]. A rapid highlight of the kidney and then of the bladder is observed (about 5 and 25 minutes after the intravenous injection of the nanoparticles respectively). A similar dose of DOTAREM® (80 μL at 40 mM in Gd) was injected in the mice and the residence time of the nanoparticles in the blood was found to be about two times higher for AGuIX than for DOTAREM® (13.2 minutes for AGuIX versus 6.8 min for DOTAREM®). This higher residence time leads to a larger imaging window for the nanoparticles in comparison to the chelates. Angiography images show a clear highlight of the blood brain vessels with a better contrast for the nanoparticles due to the higher relaxivity and the longer residence time in the blood circulation. Interestingly, the nanoparticles are eliminated from the kidney according to a multi-exponential law. The entire nanoparticles are eliminated more slowly than the partially degraded that pass directly from the kidneys to the bladder. The full nanoparticles are first taken up by renal cells of the cortex region before complete elimination.

#### On tumor bearing animals

Previous studies on brain glioma bearing rats have shown that gadolinium chelates injected intravenously can provide clear highlights of the tumors [35]. Interestingly, the contrast to noise ratio (CNR) is maximal 1–3 minutes after the injection and decreases very rapidly. For example, U. I. Attenberger *et al.* have injected GADOVIST®, DOTAREM® and MAGNEVIST® in glioma bearing rats [35]. For DOTAREM®, they obtained tumor Signal to Noise Ratio (SNR) on a 1.5 T apparatus of 30.9 ± 4.4, 54.1 ± 3.8, 51.6 ± 5.9, 45.7 ± 2.5, 44.1 ± 2.2 and 41.4 ± 1.5 (before injection and 1 min, 3 min, 5 min, 7 min and 9 min post-injection respectively). The rapid decrease in the gadolinium concentration in the tumor, which is in the same order of magnitude than the elimination time of the chelates from the blood, is problematic to determine the most suited moment for irradiation (*i.e.* higher content of gadolinium in the tissue combined to the lower one in the surrounding healthy tissues).

**Figure 2:**
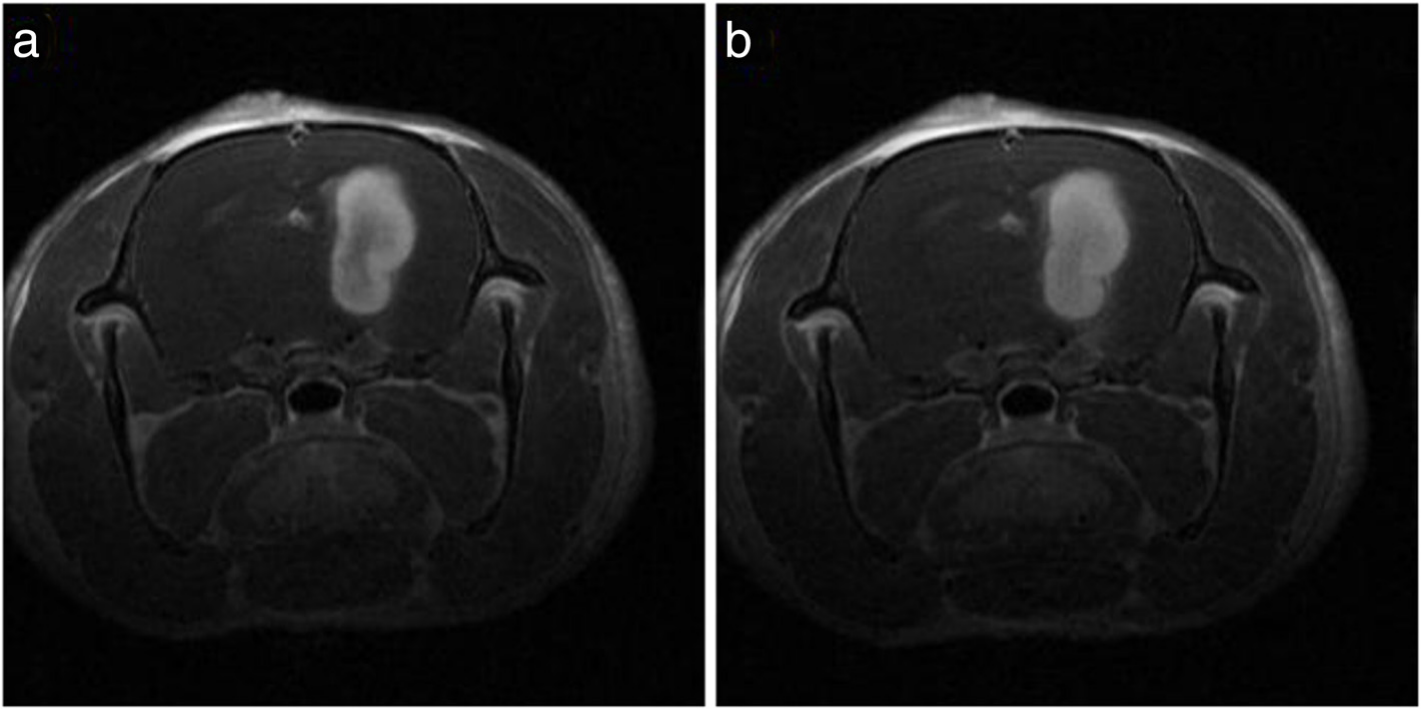
**T**_**1**_ **-weigthed images of the brain of a 9 L bearing rat: a) 7 and b) 11 minutes after intravenous injection of AGuIX nanoparticles.**

**Figure 3:**
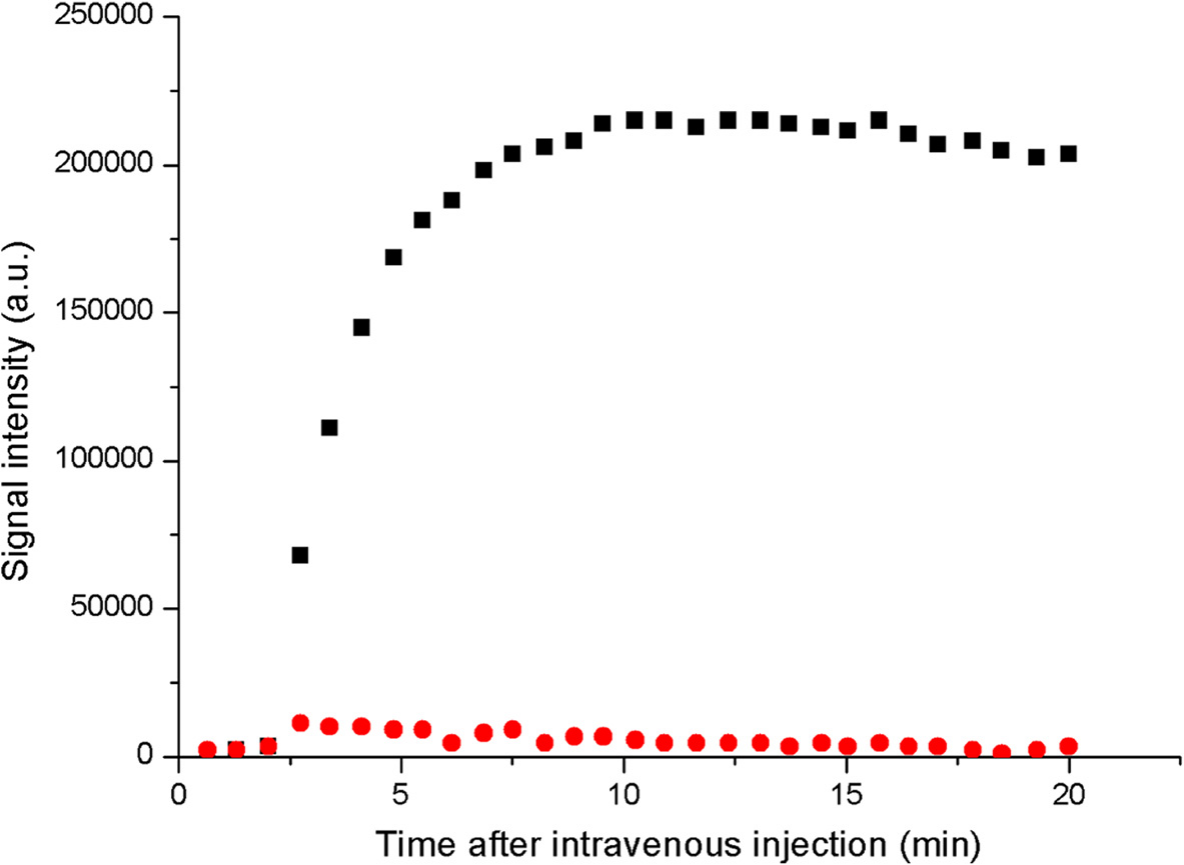
**Temporal evolution of the MRI signal in tumor (black squares) and in an equivalent surface in normal tissue in the left hemisphere (red circles).**

AGuIX nanoparticles accumulate passively in tumors thanks to the enhanced permeability and retention effect (EPR effect) [36], firstly described by Maeda *et al.* [18]. In the specific case of brain, the nanoparticles and the chelates cannot cross the blood brain barrier unless it is compromised by a pathology like a tumor. The DTPA based AGuIX nanoparticles have previously shown passive accumulation in 9LGS glioma-bearing rats [27]. This specific gliosarcoma model has been chosen due to its characteristics close to human gliomas (high proliferative capability, high vascularization and high infiltrative pattern) [33]. DOTAGA based AGuIX nanoparticles have been injected intravenously in two 9LGS glioma-bearing rats. The temporal evolutions of the T_1_ signal have been observed in both rats and are completely similar. It is then shown in Figures 2 and 3 for only one of the rats.

**Figure 4:**
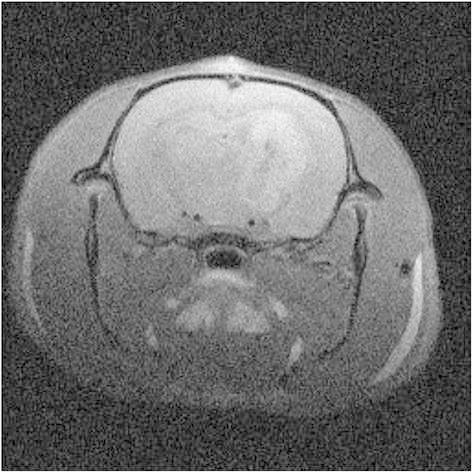
**T**_**1**_ **-weigthed images of the brain of a 9LGS bearing rat 1 day after intravenous injection of AGuIX nanoparticles.**

A small increase in the signal was observed in the healthy tissue of the rats, followed by a rapid decrease a few minutes after the injection. Conversely, a significant enhancement of the MRI signal was noticed in the tumor. It reached a plateau about 7 minutes after the intravenous injection of the nanoparticles (See Figure 3). After 1 day, a weak MRI signal can be still visualized in the tumor due to the presence of the nanoparticles (See Figure 4). The persistence of the signal is due to the very slow leakage of the nanoparticles from the tumor. The capacity of the nanoparticles to remain in the tumor even hours after their injection is a real asset to determine an adapted radiotherapy protocol. Contrary to the gadolinium chelates that present a rapid elimination from the tumor, the nanoparticles are rapidly cleared from healthy tissues while conserving a high and relatively constant concentration in the tumor.

### *In vivo* radiosensitization survival curves

**Figure 5:**
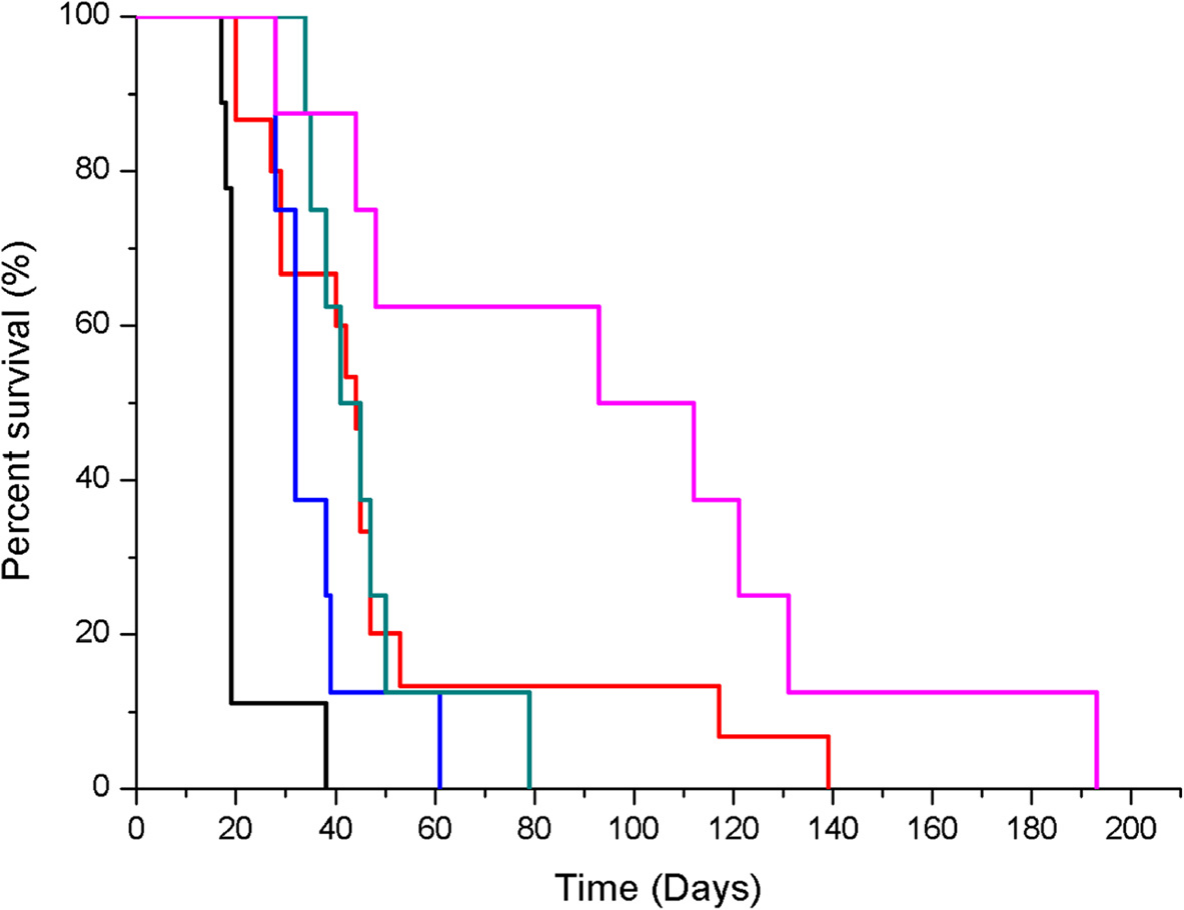
**Survival curves of 9LGS bearing rats without treatment (black curve, n = 9 rats), only treated by MRT (red curve, n = 15 rats), and treated by MRT 20 min after an injection of 1 M (56 μL) of DOTAREM® (blue curve, n = 8 rats), a unique injection of 40 mM (1.4 mL) of DOTAREM® (green curve, n = 8 rats) or AGuIX (pink curve, n = 8 rats), 10 days after tumor implantation.** The MRT irradiation was done in cross-fired mode, using 50 μm wide micro-beams, with a spacing of 200 μm between the beams. The skin entrance dose was set at 400 Gy for the peak and 20 Gy for the valley.

A comparison was performed between the efficiencies of molecular complexes, such as gadolinium chelates (DOTAREM®) and of chelates incorporated at the surface of ultrasmall nanoparticles (AGuIX), under the same irradiation conditions, *i.e.* 20 min, after intravenous injection. The short delay of 20 min has been chosen to maintain a relatively high gadolinium concentration in the tumor during irradiation when using the chelates. In a first experiment, two experimental conditions were tested for the chelates. The same quantity of gadolinium (56 μmol) was injected in all the experiments. For the chelates, two different gadolinium concentrations were used: (i) [Gd^3+^] = 1 M (injected volume of 56 μL), close from the concentrations used for DOTAREM® in clinic protocol and (ii) [Gd^3+^] = 40 mM (injected volume of 1.4 mL), the concentration used with nanoparticles. These two different concentrations were used in order to determine if the initial concentration of the solution has an influence upon the survival curves (See Figure 5). Regardless of the concentration, almost no difference is observed between the survival curves when the irradiation is performed in presence or in absence of chelates (MeST were equal to 32 and 43 days for chelates and 44 days for irradiation only) (See Table 1). These median survival times are higher than for non-treated animals (MeST of 19 days). This corresponds to an increase in lifespan (ILS) of 131%, 126% and 68% for irradiation alone or in presence of gadolinium chelates injected 20 minutes before at Gd concentrations of 40 mM and 1 M respectively.

**Table 1:** **MeST of the 9LGS bearing rats without irradiation, after MRT irradiation (i) only, (ii) in presence of DOTAREM® and (iii) in presence of particles (with the same quantity of gadolinium)**

	**Median survival time (Days)**	**Number of rats**
Without irradiation	19	9
Only MRT	44	15
MRT irradiation 20 minutes after injection of 56 μL of DOTAREM® ([Gd]^3+^ = 1 M)	32	8
MRT irradiation 20 minutes after injection of 1.4 mL of DOTAREM® ( [Gd]^3+^ = 40 mM])	43	8
MRT irradiation 20 minutes after injection of 1.4 mL of AGuIX nanoparticles ( [Gd]^3+^ = 40 mM])	102.5	8

In comparison, the irradiation of the animals 20 min after the intravenous injection of the AGuIX nanoparticles (1.4 mL and [Gd^3+^] = 40 mM) with the same amount of gadolinium than after injection of DOTAREM® leads to an important increase of the MeST to 102.5 days. It corresponds to an impressive ILS of 439%. A significant difference is found between particles and chelates associated to MRT (p < 0.0035 and p < 0.0158 for the 1 M injection and the 40 mM injection using the log rank test).

## Conclusion

To conclude, the treatment of glioma bearing rats with MRT after injection of AGuIX nanoparticles leads to an important increase in the survival of aggressive glioma bearing rats. On the contrary, no evidence of such increase has been observed with the combination of MRT and gadolinium chelates. The accumulation of particles in brain tumor is different than that of commercial chelates, presenting a fast accumulation but also a strong residence time in the tumor; they remain for hours after the injection. The plateau of MRI signal observed in the tumor, which can be linked to the gadolinium concentration, is of importance for radiotherapy as irradiation can be performed after the elimination of the nanoparticle from the blood circulation, while conserving about the same quantity of nanoparticles in the tumor. The particles allow a biodistribution that is very well suited for an optimal radiosensitization, thanks to the EPR effect previously mentioned. The particle aspect of the AGuIX is now proven to provide nanodose energy deposition in the vicinity of the nanoparticles. This deposition is due to a series of absorption/emission of Auger electrons that take place within the particles (also called Auger shower) between the Gd^3+^ cations permitting the initiation of deleterious phenomena absent with commercial gadolinium chelates. Therefore AGuIX nanoparticles present the characteristics of an adapted drug for theranostic applications: contrast enhancement for MRI due to high relaxivity, high radiosensitizing properties and adapted biodistribution (i.e. elimination by the kidneys and passive accumulation in the tumor).
